# Novel Therapeutic Strategy Based on Neutrophil Subset and Its Function in Autoimmune Disease

**DOI:** 10.3389/fphar.2021.684886

**Published:** 2021-06-07

**Authors:** Daigo Nakazawa, Takashi Kudo

**Affiliations:** Department of Rheumatology, Endocrinology, and Nephrology, Faculty of Medicine and Graduate School of Medicine, Hokkaido University, Sapporo, Japan

**Keywords:** neutrophil heterogeneity, low-density granulocyte (LDG), neutrophil extracellular traps (NETs), ANCA associated vasculitis, SLE, autoimmune disease

## Introduction

Autoimmune disease is characterized by several organ injuries due to abnormal immune responses. Disease-specific autoantibodies influence neutrophils to induce neutrophil extracellular traps (NETs) in systemic lupus erythematosus (SLE) and anti-neutrophil cytoplasmic antibody (ANCA)-associated vasculitis (AAV). The formed and excessive NETs could serve as autoantigens that produce autoantibodies against NETs and accelerate the immune response via the type I interferon signaling pathway (Gupta and Kaplan, 2016). Moreover, cytotoxic NET-components as damage-associated molecular patterns (DAMPs) injure the surrounding cells and provoke both innate and acquired immunity to exacerbate disease severity (Nakazawa et al., 2019). NETs have been associated with the development of various autoimmune diseases. However, recent studies have suggested the significance of neutrophil diversity, including phenotype, function, and development of inflammatory conditions, which also influence the characteristics of NETs (Jariwala and Laxer, 2021). In particular, human normal density neutrophils (NDNs) attempt to undergo cell lytic NETs via the stimulation of ANCA serum, leading to vascular necrosis (Kessenbrock et al., 2009; van Dam et al., 2019). On the other hand, low-density neutrophil subsets in patients with lupus, which are distinct from NDNs, form pro-inflammatory NETs leading to vascular damage (Carmona-Rivera and Kaplan, 2013), which might be involved in the development of immunological abnormalities, atherosclerosis, and thrombotic events (Carlucci et al., 2018). In this opinion article, we provided an overview of neutrophil diversity in autoimmune diseases and discussed therapeutic strategies based on the pathogenesis.

### Neutrophil Subsets and Its Function

Neutrophils are abundant white blood cells in mammals that act as innate host defenses. Peripheral neutrophils mobilize from the bone marrow to the circulation with a short life span. Mechanistically, in human and mice, the balance between CXC- chemokine receptor 2 (CXCR2) and CXCR4 regulates neutrophil mobilization and maintains an appropriate storage pool of neutrophils. Under homeostasis, surface CXCR4 on neutrophil and stromal cell-derived CXCL12 attempts to reserve as immature neutrophils in the bone marrow (De Filippo and Rankin, 2018). In bone marrow, neutrophils with reduced CXCR4 and increased CXCR2 expression are recruited into circulation as matured neutrophils (Eash et al., 2010). In circulation, aging neutrophils express CXCR4, triggering their homing back to the bone marrow and their apoptotic processes (Jaillon et al., 2020) (Furze and Rankin, 2008). As such, the mobilization and maturation occurs depending on the neutrophil-intrinsic programming and the surrounding environment. The phenotypic diversity contributes to the neutrophil physiology, including the immune response, the production of neutrophil extracellular traps (NETs), and their own clearance (Casanova-Acebes et al., 2013; Jaillon et al., 2020). In addition to the classification based on surface molecules, low-density granulocytes (LDGs) were identified as discrete population that remains in the fraction of peripheral blood mononuclear cells (PBMC) after density gradient separation. LDGs display immunosuppressive or proinflammatory properties according to the disease (Ley et al., 2018). In particular, proinflammatory LDGs are increased in autoimmune disease (SLE or psoriasis) and spontaneously undergo NETs formation (Teague et al., 2019), suggestive a role of the development of autoimmune disease and vascular injury. Although the precise physiological roles of phenotypic features of LDG subsets remain poorly understood because of the lack of their specific markers, the dysregulated LDGs might contribute to the pathology of autoimmune related vascular disease (Goel and Kaplan, 2020). AAV and SLE develop with autoantibodies against NET components, ANCAs, and anti-DNA antibodies, respectively. In human and mice, ANCAs affect normal density neutrophils (NDNs) to induce cell-lytic NETs formation, while LDGs in SLE develop spontaneous NETs. Although apoptotic neutrophils are cleared by phagocytic cells via silent immunological procedure, NETs might be insufficiently processed during destruction as a form of necrotic cells, where cytoplasmic organelles leak out and become auto-antigens (Nakazawa et al., 2012; Nakazawa et al., 2019). Future studies are necessary to understand how these different types of NETs develop and are cleared by phagocytic system in autoimmune diseases. Xie et al. (Xie et al., 2020) revealed neutrophil heterogeneity and maturation in the bone marrow, spleen and peripheral blood using mouse neutrophil single-cell RNA sequencing analysis. Neutrophils differentiate into mature types by acquiring antimicrobial capability. In the peripheral blood, three transcriptionally distinct neutrophil subsets were identified as the following. 1) The migration and inflammatory response-related genes expressing neutrophils (namely, PMNa) can arise from both mature and immature neutrophils. 2) The interferon-stimulated genes (ISGs) expressing neutrophils (PMNb) mainly arise from bone marrow mature neutrophil. 3) Relatively aging neutrophils (PMNc) are gradually developed from PMNa and PMNb. Interestingly, under microbial infectious conditions, the transforming of PMNa from immature neutrophil were suppressed and immature neutrophil predominantly differentiated into mature neutrophils, suggesting that the dynamic transition in a series of neutrophil differentiation occurs to terminate the crisis. The scRNA-seq analysis of human peripheral blood also showed similar neutrophil population including ISGs-expressing neutrophils (PMNb). Although the relationship between these three subsets and LDGs/NDNs remains unclear, this study provides a better understanding of neutrophil diversity and kinetics in autoimmune disease areas, because the LDG enrichment in SLE would be likely a consequence of increased granulopoiesis (Kegerreis et al., 2019).

### The Characteristics of LDG-NETs in SLE

SLE is characterized by systemic autoimmune disease and the presence of autoantibodies against nucleic acid-protein auto-antigens. Patients with SLE histologically show immune complexes (ICs)-mediated tissue injury underlying a chronic inflammatory response. In SLE, ICs bind to Fcγ receptors on human neutrophils to activate mitochondrial ROS, subsequently resulting in NET formation (Lood et al., 2016; van Dam et al., 2019). In human studies, the formed NETs are reportedly resistant to hemostatic clearance due to the deficiency or acquired insufficiency of deoxyribonuclease (DNase), and dysregulated NETs serve auto-antigens, producing further autoantibodies (Hakkim et al., 2010). NETs components damage organs with complement activation (Leffler et al., 2012) and induce acquired immunity via toll-like receptors (Desai et al., 2016; Nakazawa et al., 2018). During the past decade, NETs in patients with SLE occur predominantly in the LDGs subset, which displays morphologically and genetically immature characteristics and is involved in SLE development (Mistry et al., 2019). The human SLE-LDGs impair phagocytic function; instead, they show pro-inflammatory properties and injure the endothelium, indicating the involvement of cardiovascular disease development in SLE (Denny et al., 2010; Rahman et al., 2019; Bashant et al., 2021). Furthermore, the human LDGs comprise two subpopulations of CD10-negative immature and CD10-positive intermediate mature types based on transcriptomic and epigenetic analyses. In patients with SLE, the formers do not appear to have various kinds of canonical neutrophil functions, including phagocytosis, but they transcriptionally display an active phenotype, expressing cell cycle progression (Mistry et al., 2019). The latters show the high expression of type I IFN-stimulated genes and underwent NET formation with mitochondrial DNA release. These findings suggest that each subset of LDGs might be involved in the development of SLE vascular disease, via immune cell recruitment into inflammatory vascular sites and plaque instability via matrix metalloproteinase. The understanding of origin of LDGs in SLE might contribute to the elucidation of the pathogenesis. The biological analysis of gene expression and phenotypic data indicated that the enrichment of human and mice LDGs gene signature in SLE was likely to be a consequence of increased granulopoiesis, which might be caused by alternative granulopoiesis pathway (Kegerreis et al., 2019; Grigoriou et al., 2020). Further investigations of the regulatory mechanism in LDGs might offer novel therapeutic options.

### The Pathogenicity of ANCA-NETs in AAV

Anti-neutrophil cytoplasmic antibody (ANCA)-associated vasculitis (AAV) is an autoimmune disease characterized by multiple organ damage, clinically manifested by rapidly progressive glomerulonephritis and pulmonary hemorrhage. In human and mice studies, the pathogenic ANCA serum is involved in NET formation and mediated endothelial necrosis (Nakazawa et al., 2014; Watanabe-Kusunoki et al., 2020), causing necrotizing vasculitis. The formed NETs serve as autoantigens against ANCA at the site, which produces further ANCA, leading to a vicious circle (Kraaij et al., 2018). Myeloperoxidase (MPO)-ANCA binds to MPO expressed on primed human neutrophils, and the Fc region of ANCA interacts with Fcγ receptor to induce ROS production and peptidylarginine deiminase 4 (PADI4) activation, resulting in NET formation (Kusunoki et al., 2016; Watanabe-Kusunoki et al., 2020). It has been reported that ANCA predominantly influences human NDNs, forming lytic NETs with extracellular genomic DNA and cytoplasmic compositions, while human LDGs show hyposensitivity against MPO-ANCA stimulation (Ui Mhaonaigh et al., 2019). However, patients with active phase AAVs showed increased LDGs in peripheral blood, implying the possibility that the expansion might be due to the reaction of acute inflammation or similar to SLE, which acts as a pro-inflammatory mediator and is involved in disease development. Genetic analysis using bulk neutrophils in patients with AAV revealed the upregulation of MPO and PR3 transcripts; however, in the steady-state, these granule genes are only expressed on immature neutrophils in the bone marrow, not peripheral floating neutrophils after differentiation (Yang et al., 2004; Xie et al., 2020). Thus, these findings raise several questions. These immature neutrophils with the expression of primary granules are NDNs or LDGs? What is the origin of immature neutrophils from the bone marrow? or circulating mature neutrophils are re-expressed in response to the surrounding environment. How and where are these neutrophils and NETs mobilized and cleared by phagocytic cells? In line with these issues, we review the potential novel treatment in the Discussion section.

## Discussion

**FIGURE 1:**
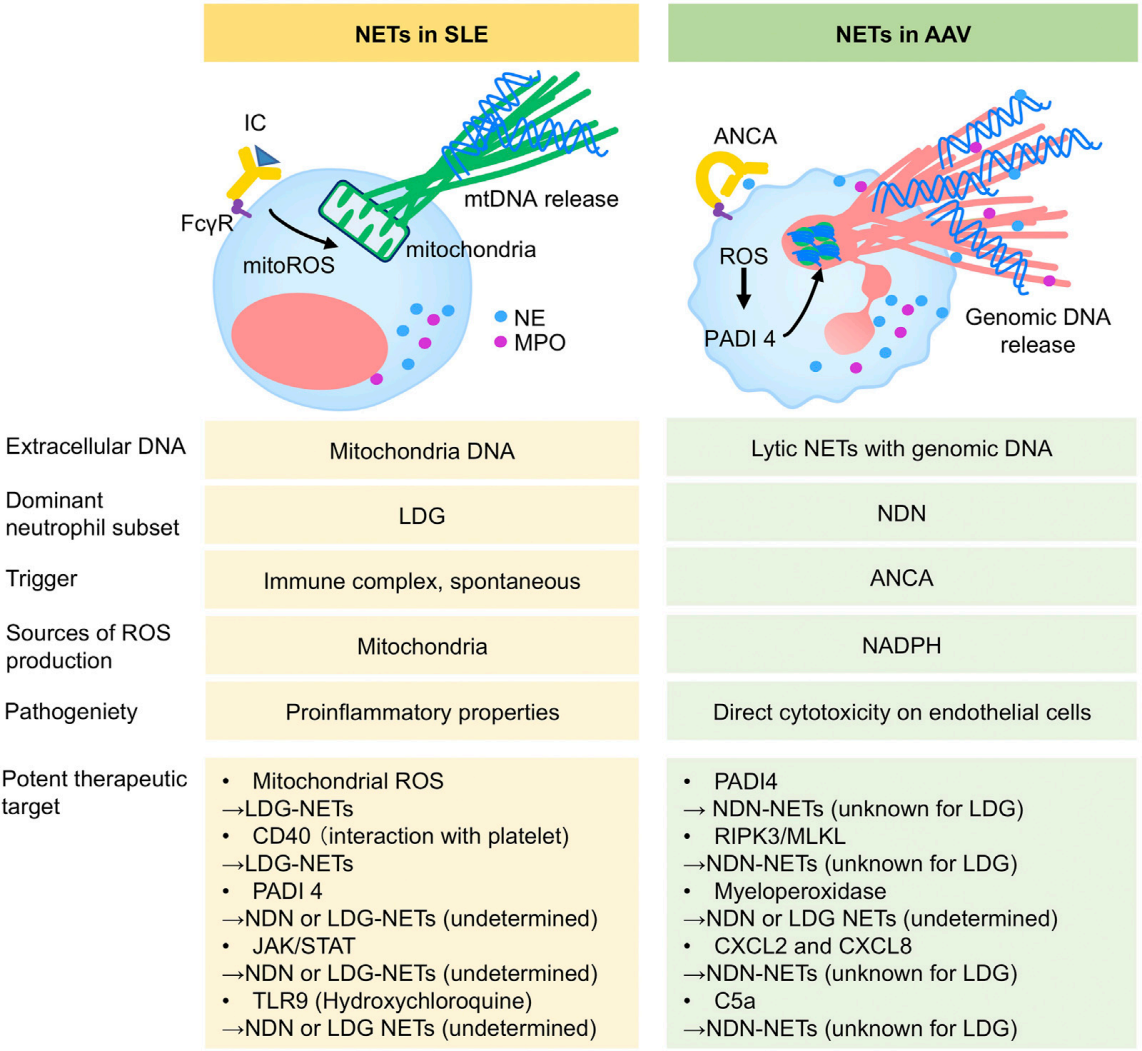
The characteristics of SLE-NETs and AAV-NETs. SLE, Systemic lupus erythematosus; AAV, ANCA, anti-neutrophil cytoplasmic antibody; AAV, ANCA-associated vasculitis; IC, immune complex; ROS, reactive oxygen species; mtROS, mitochondrial ROS; mtDNA, mitochondrial DNA, LDG, low density granulocyte; NDN, normal density neutrophil; NE, neutrophil elastase; MPO, myeloperoxidase; PADI, Peptidylarginine Deiminase; RIPK, receptor-interacting serine/threonine protein kinase; MLKL, mixed lineage kinase domain-like; JAK/STAT, Janus kinase-signal transducer and activator of transcription; TLR, toll like receptor.

Accumulating evidence supports the significance of neutrophil heterogeneity and disease-specific NETs in autoimmune diseases. The elucidation of different NET pathogenicity and signaling pathways would provide new insights into the development of therapeutic targets. The most common autoimmune diseases require glucocorticoid (GC) therapy to induce disease remission at onset/or flare and prevent relapse for a long time. However, GCs are a main cause of toxicity, including diabetes, infections, fractures, and atherosclerosis (Ruiz-Irastorza and Bertsias, 2020), leading to impaired quality of life and decreased survival in patients with autoimmune disease. In particular, cardiac involvement and chronic kidney disease underlying atherosclerosis are frequently seen in patients with SLE and AAV due to their own disease and off-target effects of GCs (Kronbichler et al., 2020). Accordingly, recent studies have focused on the challenges in replacing GCs, including the use of a sparing agent in the treatment of autoimmune diseases. Targeting neutrophils could not only provide an alternative therapeutic strategy, but also contribute to the prevention of vascular damage via the avoidance of exposure to GCs and the inhibition of pro-inflammatory neutrophils against the endothelium. In human SLE, spontaneous LDGs-NETs were suppressed by MitoTEMPO (mitochondrial superoxide scavenger) *in vitro* studies (Lood et al., 2016). The LDGs in human SLE and psoriasis interacted with activated platelet via CD40 ligand exacerbating vascular disease, suggesting the potential targeting of platelet (Duffau et al., 2010; Teague et al., 2019). In MRL/lpr lupus-prone mice, the pan-PADI inhibitor ameliorated organ injuries by reducing NETs formation (Knight et al., 2015), but the genetic deletion of PADI4 in lupus model mice (Kienhöfer et al., 2017) and human neutrophil *in vitro* studies (pan-PADI inhibition in immune complexes-mediated NETs) (van Dam et al., 2019) showed conflicting data. The role of PADI4 of NDNs and LDGs in SLE remains unclear and the targeting PADI4 should be addressed with caution. JAK inhibitor improved the disease activity of MRL/lpr lupus-prone mice via the NETs inhibition (Furumoto et al., 2017). In the clinical setting, hydroxychloroquine (HCQ) improves disease activity in patients with SLE and allows the reduction of GC dose, which could lead to the improvement of survival. These favorable findings might be due to the effect of HCQ on the regulation of NETs. Mechanistically, HCQ inhibited mice NET formation via the PADI4 and Rac2 expression by blocking toll-like receptor nine and ameliorating liver ischemia reperfusion injury in a mouse model (Zhang et al., 2020). Thus, further detailed studies are needed to elucidate how HCQ influences SLE-neutrophil heterogeneity and kinetics, which might suggest a more appropriate usage of HCQ. Of note, based on novel insights into neutrophil diversity and kinetics, targeting LDGs in SLE could become an option as a GC sparring agent and directly prevent atherosclerotic events. Similar to SLE, AAV requires a novel GC replacement strategy because intensive immunosuppressive therapy, including high-dose GCs as standard therapy for AAV, is critically relevant to adverse event-associated mortality and morbidity (Little et al., 2010) and could also increase atherosclerosis in AAV patients. In terms of NET control, human and mice studies indicate that the inhibition of PADI4 (Kusunoki et al., 2016; van Dam et al., 2019) and receptor-interacting serine/threonine protein kinase (RIPK) three signlaing (Schreiber et al., 2017; van Dam et al., 2019) could be a therapeutic candidate via NDN-NETs inhibition. Based on experimental animal studies, the inhibition of chemoattractants such as CXCL2 and CXCL8 might lead to the resolution of inappropriate neutrophil mobilization and NETs induction, leading to the improvement of vasculitis and atherosclerosis (Summers et al., 2010; Kanzaki et al., 2016; An et al., 2019). Human histological findings in patients with AAV revealed the involvement of extracellular myeloperoxidase in glomerular injury (O'Sullivan et al., 2015). The inhibitor of complement 5a (C5a) receptor, whose effect is shown in human (clinical phase three trial (Jayne et al., 2021)) and mouse study (Xiao et al., 2014), directly affects NDNs to inhibit C5a mediated-neutrophil priming and activation, inducing clinical remission. Elucidating the mechanistic effects of C5a on heterogeneous neutrophil/NET biology may provide a better understanding and convincing evidence. Taken together, unraveling neutrophil physiology and NETs characteristics could help establish novel strategies and address the unmet needs of autoimmune disease (Figure 1).
